# Replication Study for the Association of Seven Genome- Gwas-Identified Loci With Susceptibility to Ovarian Cancer in the Polish Population

**DOI:** 10.1007/s12253-014-9822-6

**Published:** 2014-08-31

**Authors:** Adrianna Mostowska, Stefan Sajdak, Piotr Pawlik, Janina Markowska, Monika Pawałowska, Margarita Lianeri, Paweł P. Jagodzinski

**Affiliations:** 1 Department of Biochemistry and Molecular Biology, Poznań University of Medical Sciences, 6 Święcickiego St., 60-781 Poznań, Poland; 2 Clinic of Gynecological Surgery, Poznań University of Medical Sciences, Poznań, Poland; 3 Chair of Gynecologic Oncology, Poznań University of Medical Sciences, Poznań, Poland

**Keywords:** Ovarian cancer, Single nucleotide polymorphisms, Genome-wide association studies

## Abstract

We investigated the previously-demonstrated association of seven genome-wide association studies (GWAS) single nucleotide polymorphisms (SNPs), including rs2072590 (*HOXD-AS1*), rs2665390 (*TIPARP*), rs10088218 and rs10098821 (8q24), rs3814113 (9p22), rs9303542 (*SKAP1*) and rs2363956 (*ANKLE1*), as risk factors of epithelial ovarian tumors (EOTs). These SNPs were genotyped in two hundred seventy three patients with EOTs and four hundred sixty four unrelated healthy females from the Polish population. We observed the lowest p values of the trend test for the 9p22 rs3814113 and 8q24 rs10098821 SNPs in patients with all subtypes of ovarian cancer (p_trend_ = 0.010 and p_trend_ = 0.014, respectively). There were also significant p values for the trend of the 9p22 rs3814113 and the 8q24 rs10098821 SNPs for serous histological subtypes of ovarian cancer (p_trend_ = 0.006, p_trend_ = 0.033, respectively). Moreover, stratification of the patients based on their histological type of cancer demonstrated, in the dominant hereditary model, a significant association of the 9p22 rs3814113 SNP with serous ovarian carcinoma OR = 0.532 (95 % CI = 0.342 - 0.827, *p* = 0.005, p_corr_ = 0.035). Despite the relatively small sample size of cases and controls, our studies confirmed some of the previously-demonstrated GWAS SNPs as genetic risk factors for EOTs.

**Electronic supplementary material:**

The online version of this article (doi:10.1007/s12253-014-9822-6) contains supplementary material, which is available to authorized users.

## Introduction

Epithelial ovarian tumors (EOTs) are currently the leading cause of mortality among gynecological carcinomas in Europe and the United States, causing approximately 4 % of deaths from malignancies in women [1, 2]. This high mortality of EOTs is due to late diagnosis, which results from the nonspecific symptoms in the beginning stages of EOTs and a lack of robust serum biomarkers for EOTs screening [3]. There are recognized factors that can either reduce or increase the risk of EOTs development [4–16]. Multiparity, breastfeeding, tubal ligation and oral contraceptive use all display a protective role in ovarian cancer development [4–8]. The risk factors for EOTs include early age of menarche, late age of natural menopause, hormone replacement therapy (HRT), nulliparity , infertility, obesity and some lifestyle factors [8–13]. Other factors contributing to EOTs development include endometriosis, pelvic inflammatory disease, environmental toxins and geographical location, the latter related to sun exposure and vitamin D production [13–16]. However, one of the greatest risk factors for EOTs are inherited genetic components, including a family history of ovarian tumors, especially in first-degree relatives, and a personal history of breast tumors [17–21]. The firmly established genetic background of EOTs encompasses certain high-penetrance genes: *BRCA1* (3–6 %), *BRCA2* (1–3 %), and *HNPCC* DNA mismatch repair genes (1–2 %) [19–21]. However, the genetic variants of high-penetrance genes are involved in less than 40 % of the hereditary susceptibility to EOTs [19–21]. This suggests that the development of EOTs may involve low-penetrance risk genes that may account for a variable heritability pattern. in a multigenic EOTs model [19–21]. The early events and pathogenesis of ovarian tumorigenesis remain elusive [21]. Three recently conducted genome-wide association studies (GWAS) in patients with EOTs indicated seven risk alleles amounting genome-wide significance, at loci 9p22, 8q24, 2q31, 19p13, 3q25 and 17q21 [22–24]. We replicated the distribution of the top seven ovarian cancer susceptibility GWAS SNPs including rs2072590 on 2q31 (*HOXD-AS1*), rs2665390 on 3q25 (*TIPARP*), rs10088218 and rs10098821 on 8q24, rs3814113 on 9p22, rs9303542 on 17q21 (*SKAP1*) and rs2363956 on 19p13 (*ANKLE1*), in patients with ovarian cancer and controls from a sample of the Polish population.

## Material and Methods

### Patients and Controls

The patient group consisted of 273 women with histologically diagnosed ovarian carcinoma according to the International Federation of Gynecology and Obstetrics (FIGO). They were enrolled into the study from the University Hospital, Clinic of Gynecological Surgery and Chair of Gynecologic Oncology at Poznan University of Medical Sciences. Histopathological classification, describing the stage, grade and tumor type, was carried out by an experienced pathologist (Table 1). The controls included 464 unrelated healthy female volunteers who were matched by age to the cancer patients (Table 1). The patients and healthy female volunteers were Caucasian from the Wielkopolska area of Poland. Written informed consent was obtained from all participating individuals. The study design was accepted by the Local Ethical Committee of Poznań University of Medical Sciences.

**Table 1:** Clinical characteristics of ovarian cancer patients and healthy controls

Characteristic	Patients	Controls
	(*n* = 273)	(*n* = 464)
Mean age ± SD	53.9 ± 9.1	52.8 ± 8.2
Histological grade		
G1	81 (29.7 %)	
G2	101 (37.0 %)	
G3	91 (33.3 %)	
Gx	0 (0.0 %)	
Clinical stage		
I	104 (38.1 %)	
II	43 (15.8 %)	
III	91 (33.3 %)	
IV	35 (12.8 %)	
Histological type		
Serous	97 (35.5 %)	
Mucinous	30 (11.0 %)	
Endometrioid	53 (19.4 %)	
Clear cell	26 (9.5 %)	
Brenne	0 (0.0 %)	
Mixed	24 (8.8 %)	
Solid	18 (6.6 %)	
Untyped carcinoma	25 (9.2 %)	

### Genotyping

Genomic DNA was obtained from peripheral blood leucocytes by salt extraction. DNA samples were genotyped for the seven SNPs: intronic rs2072590 on 2q31 (*HOXD-AS1*)*,* intronic rs2665390 on 3q25 (*TIPARP*), rs10088218 and rs10098821 on 8q24, rs3814113 on 9p22, intronic rs9303542 on 17q21 (*SKAP1*) and missense rs2363956 on 19p13 (Leu184Trp, *ANKLE1*) (Supplemental Table 1). SNPs were selected based on the highest association in GWAS studies [22–24]. Genotyping of the *HOXD-AS1* rs2072590, *TIPARP* rs2665390, 8q24 rs10088218 and rs10098821, *SKAP1* rs9303542 and *ANKLE1* rs2363956 was performed by high resolution melting curve analysis (HRM) on the LightCycler 480 system (Roche Diagnostics, Mannheim, Germany (Supplemental Table 2). Genotyping of the 9p22 rs3814113 SNP was performed by PCR, followed by appropriate restriction enzyme digestion (PCR-RFLP) according to the manufacturer’s instructions (Fermentas, Vilnius, Lithuania). Primer sequences and conditions for HRM and PCR-RFLP analyses are presented in Supplemental Table 2. Genotyping quality was assessed by commercial sequencing of approximately 10 % randomly selected samples.

### Statistical Analysis

Hardy-Weinberg equilibrium (HWE) was evaluated by Pearson’s goodness-of-fit Chi-squared (χ^2^) statistic. The data were tested for association with ovarian cancer using the Cochran-Armitage trend test. The distinction in the allele and genotype frequencies between cancer patients and healthy female volunteers were determined using standard χ^2^ or Fisher tests. The odds ratio (OR) and associated 95 % confidence intervals (95%CI) were also calculated. SNPs were assessed under recessive and dominant inheritance models. To adjust for the multiple testing, we used a Bonferroni correction. High order gene-gene interactions among all tested polymorphic loci were evaluated by the multifactor dimensionality reduction (MDR) approach (MDR version 2.0 beta 5) [25]. Based on the obtained testing balanced accuracy and cross-validation consistency values, the best statistical gene-gene interaction models were established. A 1000*-*fold permutation test was used to assess the statistical significance of MDR models (MDR permutation testing module 0.4.9 alpha).

## Results

### Contribution of rs2072590 (*HOXD-AS1*)*,* rs2665390 (*TIPARP),* rs10088218 and rs10098821 (8q24)*,* rs3814113 (9p22)*,* rs9303542 (*SKAP1*) and rs2363956 (*ANKLE1*) SNPs to Ovarian Cancer Development

The prevalence of *HOXD-AS1, TIPARP,* 8q24*,* 9p22*, SKAP1* and *ANKLE1* genotypes did not display deviation from HWE between the patient and control groups (*p* > 0.05). The number of genotypes, OR, and 95 % CI values for the seven *HOXD-AS1, TIPARP,* 8q24*,* 9p22*, SKAP1* and *ANKLE1* polymorphisms are presented in Table 2. The lowest p values of the trend test in patients with all histological EOT subtypes were found for the 9p22 rs3814113 and 8q24 rs10098821 SNPs (p_trend_ = 0.010 and p_trend_ = 0.014, respectively) (Table 2). Moreover, we observed significant p values of the trend for the 9p22 rs3814113 and 8q24 rs10098821SNPs for serous histological subtypes of ovarian cancer (p_trend_ = 0.006 and p_trend_ = 0.033, respectively) (Table 2).

**Table 2:** Associations of nucleotide variants identified by GWAS with the risk of ovarian cancer

Chr	rs no.	Alleles^a^	MAF^b^	Genotypes cases^c^		Genotypes controls^c^	p_genotypic_ value	p_trend_ value	p_allelic_ value	OR_dominant_ (95 % CI)^d^; p value	OR_recessive_(95 % CI)^e^; p value
**2q31**	**rs2072590**	**G / t**	**0.35**	**All**	**116 / 115 / 41**	**198 / 207 / 59**	0.633	0.652	0.686	1.001 (0.740–1.355); 0.995	1.218 (0.793–1.873); 0.368
				Serous	38 / 43 / 16		0.579	0.343	0.379	1.156 (0.739–1.808); 0.526	1.356 (0.743–2.475); 0.320
				Mucinous	11 / 16 / 3		0.644	0.797	0.905	1.286 (0.598–2.764); 0.519	0.763 (0.224–2.594); 1.000^f^
				Endometrioid	28 / 15 / 10		0.067	0.690	0.762	0.665 (0.376–1.175) ; 0.159	1.596 (0.761–3.348); 0.212
				Clear cell	9 / 11 / 6		0.301	0.183	0.230	1.406 (0.614–3.221); 0.418	2.059 (0.794–5.338); 0.130
				Mixed	11 / 10 / 3		0.952	0.813	0.933	0.880 (0.386–2.005); 0.760	0.981 (0.284–3.390); 1.000^f^
				Solid tumor	7 / 9 / 1		0.645	0.750	0.891	1.063 (0.398–2.843); 0.903	0.429 (0.056–3.297); 0.708^f^
				Untyped	12 / 11 / 2		0.748	0.471	0.566	0.806 (0.360–1.806); 0.600	0.597 (0.137–2.598); 0.756^f^
**3q25**	**rs2665390**	**c / T**	**0.09**	**All**	**218 / 50 / 2**	**380 / 77 / 5**	0.744	0.715	0.784	1.105 (0.742–1.625); 0.610	0.682 (0.131–3.542); 1.000^f^
				Serous	75 / 20 / 1		0.619	0.389	0.463	1.298 (0.756–2.226); 0.343	0.962 (0.111–8.333); 1.000^f^
				Mucinous	27 / 3 / 0		0.522	0.255	0.354^f^	0.515 (0.153–1.739); 0.452^f^	N/A
				Endometrioid	43 / 8 / 0		0.740	0.606	0.734	0.862 (0.391–1.903); 0.713	N/A
				Clear cell	20 / 5 / 1		0.425	0.344	0.471	1.390 (0.541–3.571); 0.492	3.656 (0.411–32.504); 0.281^f^
				Mixed	17 / 7 / 0		0.261	0.240	0.082	1.908 (0.766–4.751); 0.159	N/A
				Solid tumor	15 / 3 / 0		0.906	0.829	0.400^f^	0.818 (0.234–2.856); 1.000^f^	N/A
				Untyped	21 / 4 / 0		0.867	0.740	0.791	0.883 (0.295–2.641); 1.000^f^	N/A
**8q24**	**rs10088218**	**a / G**	**0.12**	**All**	**223 / 44 / 4**	**357 / 100 / 7**	0.214	0.120	0.137	0.718 (0.491–1.050); 0.086	0.978 (0.284–3.373); 1.000^f^
				Serous	80 / 16 / 0		0.249	0.118	0.152	0.667 (0.374–1.190); 0.168	N/A
				Mucinous	26 / 4 / 0		0.427	0.193	0.301^f^	0.513 (0.175–1.504); 0.265^f^	N/A
				Endometrioid	42 / 11 / 0		0.655	0.566	0.679	0.874 (0.435–1.757); 0.705	N/A
				Clear cell	21 / 4 / 1		0.519	0.874	0.873	0.794 (0.293–2.158); 0.812^f^	2.611 (0.309–22.066); 0.356^f^
				Mixed	19 / 3 / 2		0.036	0.642	0.805	0.878 (0.320–2.408); 1.000^f^	5.935 (1.164–30.263); 0.068^f^
				Solid tumor	15 / 2 / 0		0.531	0.260	0.418^f^	0.445 (0.100–1.977); 0.383^f^	N/A
				Untyped	20 / 4 / 1		0.529	0.953	0.952	0.834 (0.306–2.276); 1.000^f^	2.720 (0.321–23.020); 0.345
**8q24**	**rs10098821**	**C / t**	**0.11**	**All**	**233 / 34 / 3**	**363 / 94 / 6**	0.028	**0.014**	0.016	0.576 (0.382–0.870); 0.008	0.856 (0.212–3.451); 1.000^f^
				Serous	84 / 12 / 0		0.099	**0.033**	0.045	0.519 (0.272–0.988); 0.043	N/A
				Mucinous	25 / 4 / 0		0.558	0.285	0.390^f^	0.581 (0.196–1.708); 0.481^f^	N/A
				Endometrioid	44 / 9 / 0		0.583	0.358	0.451	0.743 (0.351–1.576); 0.435^f^	N/A
				Clear cell	25 / 1 / 0		0.093	0.033	0.036^f^	0.145 (0.019–1.085); 0.025^f^	N/A
				Mixed	19 / 3 / 2		0.023	0.514	0.667	0.955 (0.348–2.623); 1.000^f^	6.924 (1.321–36.304); 0.054^f^
				Solid tumor	16 / 1 / 0		0.293	0.122	0.165^f^	0.227 (0.030–1.732); 0.141^f^	NA
				Untyped	20 / 4 / 1		0.488	0.905	0.905	0.908 (0.332–2.479); 1.000^f^	3.174 (0.367–27.435); 0.310^f^
**9p22**	**rs3814113**	**c / T**	**0.41**	**All**	**123 / 114 / 35**	**167 / 213 / 82**	0.033	**0.010**	0.009	0.696 (0.505–0.930); 0.015	0.684 (0.446–1.050); 0.081
				Serous	50 / 36 / 11		0.016	**0.006**	0.006	**0.532 (0.342–0.827); 0.005**	0.593 (0.303–1.160); 0.123
				Mucinous	12 / 12 / 6		0.809	0.905	0.903	0.849 (0.399–1.806); 0.671	1.159 (0.459–2.925); 0.755
				Endometrioid	19 / 24 / 9		0.996	0.936	0.935	0.983 (0.542–1.784); 0.956	0.970 (0.455–2.068); 0.937
				Clear cell	12 / 11 / 3		0.524	0.257	0.310	0.661 (0.299–1.461); 0.303	0.605 (0.177–2/062); 0.596^f^
				Mixed	9 / 12 / 3		0.800	0.656	0.762	0.944 (0.404–2.203); 0.893	0.662 (0.193–2.272); 0.782^f^
				Solid tumor	10 / 7 / 1		0.178	0.064	0.085	0.453 (0.175–1.170); 0.094	0.273 (0.036–2.078); 0.335^f^
				Untyped	11 / 12 / 2		0.422	0.226	0.277	0.721 (0.320–1.623); 0.427	0.403 (0.093–1.744); 0.282^f^
**17q21**	**rs9303542**	**A / g**	**0.25**	**All**	**134 / 123 / 15**	**261 / 175 / 28**	0.134	0.162	0.193	1.324 (0.981–1.788); 0.067	0.909 (0.476–1.734); 0.772
				Serous	54 / 36 / 6		0.997	0.975	0.975	1.000 (0.642–1.558); 1.000	1.038 (0.418–2.581); 0.936
				Mucinous	17 / 12 / 1		0.823	0.785	0.907	0.983 (0.467–2.072); 0.964	0.537 (0.071–4.089); 1.000^f^
				Endometrioid	24 / 26 / 3		0.270	0.230	0.285	1.554 (0.876–2.751); 0.128	0.934 (0.274–3.185); 1.000^f^
				Clear cell	12 / 11 / 3		0.414	0.207	0.274	1.500 (0.679–3.314); 0.313	2.031 (0.575–7.179); 0.222^f^
				Mixed	9 / 15 / 0		0.039	0.315	0.413	2.143 (0.919–4.997); 0.072	N/A
				Solid tumor	9 / 9 / 0		0.388	0.988	0.988	1.286 (0.501–3.299); 0.600	N/A
				Untyped	9 / 14 / 2		0.138	0.076	0.112	2.286 (0.990–5.280); 0.047	1.354 (0.304–6.038); 0.660^f^
**19p13**	**rs2363956**	**G / t**	**0.49**	**All**	**56 / 154 / 62**	**115 / 244 / 105**	0.196	0.399	0.451	1.271 (0.885–1.825); 0.193	1.009 (0.706–1.443); 0.959
				Serous	17 / 53 / 26		0.235	0.139	0.169	1.531 (0.870–2.694); 0.137	1.270 (0.770–2.094); 0.348
				Mucinous	7 / 18 / 5		0.559	0.738	0.837	1.083 (0.453–2.590); 0.858	0.684 (0.255–1.831); 0.650^f^
				Endometrioid	13 / 28 / 12		0.948	0.971	0.979	1.104 (0.524–1.962); 0.967	1.001 (0.507–1.974); 0.998
				Clear cell	8 / 11 / 7		0.719	0.911	0.906	0.741 (0.314–1.751); 0.493	1.260 (0.515–3.079); 0.612
				Mixed	6 / 17 / 1		0.059	0.203	0.265	0.989 (0.383–2.551); 0.981	0.149 (0.020–1.114); 0.039^f^
				Solid tumor	1 / 12 / 5		0.145	0.146	0.206	5.602 (0.737–42.578); 0.088^f^	1.315 (0.458–3.774); 0.574^f^
				Untyped	4 / 15 / 6		0.512	0.476	0.579	1.730 (0.582–5.146); 0.473^f^	1.080 (0.420–2.774); 0.873

*N/A* not applicable

Statistically significant results for dominan and recessive model are highlighted in bold (*p* < 0.00714)

^a^Uppercase denotes the more frequent allele in the control samples

^b^MAF, minor allele frequency calculated from the control samples

^c^The order of genotypes: DD / Dd / dd (d is the minor allele in the control samples)

^d^Dominant model: dd + Dd vs DD (d is the minor allele)

^e^Recessive model: dd vs Dd + DD (d is the minor allele)

^f^Fisher exact test

The statistical significance for multiple testing determined by correction of gene number was *p* = 0.007. Therefore, none of the seven *HOXD-AS1, TIPARP ,*8q24*,* 9p22*, SKAP1,*and *ANKLE1* polymorphisms displayed a significant association with all subtypes of ovarian cancer either in dominant or recessive inheritance models (Table 2). Stratification of the patients based on histological type of cancer revealed, in the dominant hereditary model, a significant association of the 9p22 rs3814113 SNP with serous ovarian carcinoma, OR = 0.532 (95 % CI = 0.342 - 0.827, *p* = 0.005). However, the 9p22 rs3814113 polymorphism did not display significant association with other histological types and any histological grade and clinical stage. Furthermore, there was no significant association between the *HOXD-AS, TIPARP*, *8q24, SKAP1* and *ANKLE1* polymorphisms with clinical stage, histological grade and subtype.

### MDR Analysis of Gene-gene Interactions among the rs2072590 (HOXD-AS1), rs2665390 (TIPARP), rs10088218 and rs10098821 (8q24), rs3814113 (9p22), rs9303542 (SKAP1) and rs2363956 (ANKLE1) SNPs

Exhaustive MDR analysis assessing two- to four-loci combinations of all studied SNPs for each comparison did not reveal statistical significance in predicting susceptibility to EOTs development (Table 3). The best combination of possibly interactive polymorphisms was observed for 8q24 rs10098821 and 9p22 rs3814113 (testing balanced accuracy = 0.516 %, cross validation consistency of 3 out of 10, permutation test *p* = 0.682).

**Table 3:** Results of gene-gene interactions analyzed by MDR method

Polymorphisms	Testing balanced accuracy	Cross validation consistency	p value^a^
8q24_rs10098821, 9p22_rs3814113	0.516	60 %	0.682
8q24_rs10098821, 9p22_rs3814113, 17q21_rs9303542	0.514	40 %	0.708
2q31_rs2072590, 9p22_rs3814113, 17q12_rs757210, 19p13_rs2363956	0.507	70 %	0.783

^a^Significance of accuracy (empirical p value based on 1,000 permutations)

## Discussion

Family and twin investigations have provided us with concrete evidence indicating that there are inherited genetic factors involved in susceptibility to the development of EOTs [17, 18]. GWAS have been performed in order to identify common low-penetrance ovarian cancer susceptibility genes [22–24]. The GWAS conducted by Song et al*.* (2009) demonstrated the 9p22 rs3814113 SNP to be a significant genetic risk factor contributing to all histological subtypes of EOTs [22]. In addition to this finding, GWAS analysis performed by Goode et al. (2010) found genome-wide significant association for the 3q25 rs2665390, 17q21 rs9303542, 8q24 rs10088218 and 2q31 rs2072590 SNPs with all EOTs subtypes [23]. The GWAS by Bolton et al. (2010) demonstrated that SNPs rs8170 and rs2363956 on 19p13 displayed genome-wide significance for susceptibility of serous ovarian cancer but not all histological subtypes of EOTs [24].

Our follow-up studies, conducted in Caucasian women with ovarian cancer enrolled in the Wielkopolska area of Poland, identify a significant p trend of rs3814113 on 9p22 with all sybtypes of EOTs. In addition to this finding, we observed that rs3814113 on 9p22 may play a protective role from the development of serous histological subtypes of ovarian carcinoma. The stratification of the GWAS by Song et al. (2009) that was based on histological subtypes also indicated that rs3814113 exhibited the greatest association with serous subtypes of EOTs [22]. Moreover, the 9p22 rs3814113 SNP has been demonstrated to be a protective genetic factor of ovarian cancer for carriers of *BRCA1* or *BRCA2* mutations [26]. There has also been a recent evaluation of the functional role of seven ovarian cancer susceptibility GWAS polymorphisms in association with microRNAs (miRNAs) presence [27]. This study demonstrated the highest numbers of miRNAs, 68 significantly linked to the rs3814113 SNP [27]. Moreover, the rs3814113 polymorphism was significantly associated with miR-17–92 cluster, which is considered the most remarkable cluster involved in tumorigenesis [27]. Additionally, cell carriers of the rs3814113 SNP displayed prominence of several elementary biological pathways such as cellular response to stress, adenyl nucleotide binding, intracellular organelle lumen, and others [27]. Other functional studies assessed the relationship between changeability of gene expression and the presence of seven ovarian cancer susceptibility GWAS SNPs [28]. These studies demonstrated significant association between the 9p22 rs3814113 SNP and changes in the levels of 274 mRNAs [28]. However, the strongest association of the rs3814113 SNP was observed for increased levels of *MT1G* and *ATL2* mRNAs, which respectively encode metallothionein 1G (OMIM *156353) and atlastin GTPase (OMIM *609368) [28].

Our studies also found significant p trend values for the 8q24 rs10098821 SNP for all patients with ovarian cancer, and also specifically for serous histological subtype. The Goode et al. (2010) GWAS analysis also demonstrated a generally greater association of the 8q24 rs10098821 SNPs with serous as compared to other ovarian EOTs subtypes [23]. Moreover, the 8q24 locus was found to be a risk for several malignancies encompassing breast, prostate, and colorectal cancer [29, 30]. A functional association study between GWAS SNPs and whole genome mRNA expression profiles revealed that the 8q24 rs10098821 SNP had the largest number of significant associations, specifically 38 [28]. The study also indicated possible cis-associations between rs10098821 and *MYC* expression [28]. The 8q24 polymorphisms linked to EOTs and other carcinomas are situated approximately 700 kb 3′ of the *MYC* protooncogene, and these SNPs probably control the expression of this oncogene distally [28, 31].

Presently, genetic risk evaluation for ovarian cancer can be conducted for subjects with a family history of some cancer and/or *BRCA1/2* mutations identified within families. However, the usage of low-penetrance SNPs in screening for the risk of ovarian cancer in various ethnicities has not yet been employed. This is in contrast to colorectal and breast cancers, where combinations of low penetrance risk genetic variants are already employed for susceptibility screening in some populations [32, 33].

It was demonstrated that the 9p22 rs3814113 and 8q24 rs10098821 variants were associated with the risk of EOTs in subjects of European ancestry [22, 23]. In the subjects of non-European ancestry (African or Asian ethnic group), these SNPs did not show statistically significant correlations with the risk of EOTs; however, these results could be due to small sample size [22]. Our study found a significant association of the 9p22 rs3814113 SNP with serous subtypes, and significant trend p-values for the 9p22 rs3814113 and 8q24 rs10098821 SNPs with all EOTs and serous subtypes in Caucasian patients from the Wielkopolska region of Poland. However, our replication studies have been conducted in relatively small patient and control groups, resulting in a possible missed significant association for the other studied SNPs in ovarian cancer. Therefore, this study should be replicated in other independent cohorts to validate the role of low penetrance SNPs in EOTs development and also in their use as screening tools in the evaluation of ovarian cancer susceptibility.

## Electronic supplementary material

Below is the link to the electronic supplementary material.

ESM 1 (DOCX 14 kb)

ESM 2 (DOCX 17 kb)
