# Supplementary material for: Replication Study for the Association of Seven Genome- Gwas-Identified Loci With Susceptibility to Ovarian Cancer in the Polish Population
Source: Pathol Oncol Res. 2014 Aug 31;21(2):307–13. doi: 10.1007/s12253-014-9822-6 (PMC4422849; doi:10.1007/s12253-014-9822-6)
Supplement: Supplementary file 1 — (DOCX 14 kb) [file 12253_2014_9822_MOESM1_ESM.docx]

**Supplementary Table 1. Characteristics of Polymorphisms Genotyped in the Data Set.**

| **Chromosome** | **rs no.** | **Location** | **SNP function^a^** | **MAF^b^** |
| --- | --- | --- | --- | --- |
| **2q31** | **rs2072590** | chr2:177042633 | intronic (gene: *HOXD-AS1*) | 0.35 |
| **3q25** | **rs2665390** | chr3:156397749 | intronic (gene: *TIPARP,* OMIM: *612480) | 0.09 |
| **8q24** | **rs10088218** | chr8:129543949 | N/A | 0.12 |
| **8q24** | **rs10098821** | chr8:129559228 | N/A | 0.11 |
| **9p22** | **rs3814113** | chr9:16915021 | N/A | 0.41 |
| **17q21** | **rs9303542** | chr17:46411500 | intronic (gene: *SKAP1,* OMIM: *604969) | 0.25 |
| **19p13** | **rs2363956** | chr19:17394124 | missense: Leu184Trp (gene: *ANKLE1*) | 0.49 |

^a^According to the Single Nucleotide Polymorphism database (dbSNP).

^b^MAF, minor allele frequency calculated from the control samples.
