# Supplementary material for: Replication Study for the Association of Seven Genome- Gwas-Identified Loci With Susceptibility to Ovarian Cancer in the Polish Population
Source: Pathol Oncol Res. 2014 Aug 31;21(2):307–13. doi: 10.1007/s12253-014-9822-6 (PMC4422849; doi:10.1007/s12253-014-9822-6)
Supplement: Supplementary file 2 — (DOCX 17 kb) [file 12253_2014_9822_MOESM2_ESM.docx]

**Supplementary Table 2.**

**HRM and RFLP Conditions for the Identification of Polymorphisms Genotyped in the Data Set.**

|  |  |  |  |  |  |  | **HRM analysis^c^** | **RFLP analysis^d^** | |
| --- | --- | --- | --- | --- | --- | --- | --- | --- | --- |
| **Chr.** | **rs no.** | **Location^a^** | **Alleles^b^** | **Primers for PCR amplification  (5’ – 3’)** | **Annealing temp. (°C)** | **PCR product length (bp)** | **Melting temp. range (°C)** | **Restriction enzyme** | **Restriction fragment length (bp)** |
| **2q31** | **rs2072590** | chr2:177042633 | G / t | F: TACCATATCCGTCCCCACCT | 60.6 | 101 | 80 - 90 |  |  |
|  |  |  |  | R: GAGGGGGATTGAGGGAAGAT |  |  |  |  |  |
| **3q25** | **rs2665390** | chr3:156397749 | c / T | F: GCATTACAGAAACATGGTT | 55.4 | 84 | 70 - 80 |  |  |
|  |  |  |  | R: ACCATTTACCCATTACTGA |  |  |  |  |  |
| **8q24** | **rs10088218** | chr8:129543949 | a / G | F: CTCCTGGGACAAATGTTTCG | 60.6 | 153 | 79 - 89 |  |  |
|  |  |  |  | R: CTTGTGAGTTTGGCACCTGA |  |  |  |  |  |
| **8q24** | **rs10098821** | chr8:129559228 | C / t | F: GTGAATGAAGAAACTAGAGGTG | 60.6 | 150 | 72 - 82 |  |  |
|  |  |  |  | R: AAGATATTAATCCTTTACTTTATGATG |  |  |  |  |  |
| **9p22** | **rs3814113** | chr9:16915021 | c / T | F: CATACAGCTCGTGACCTTGG | 66.3 | 267 |  | MboII | C = 267 |
|  |  |  |  | R: TTCGTCCAGCTAAAGTGTGC |  |  |  |  | T = 147 + 120 |
| **17q21** | **rs9303542** | chr17:46411500 | A / g | F: AGGTACTCAGATGCAGTTTC | 60.6 | 96 | 74 - 84 |  |  |
|  |  |  |  | R: AGCTATAAGAAGGTGTCAGG |  |  |  |  |  |
| **19p13** | **rs2363956** | chr19:17394124 | G / t | F: TCCATAGCACTCCAAAAGCAG | 60.6 | 114 | 80 - 90 |  |  |
|  |  |  |  | R: CCCATGTTTGTCCACAGTTTC |  |  |  |  |  |

^a^Based on UCSC Human Genome Browser, February 2009 human reference sequence (GRCh37)

^b^Uppercase denotes the more frequent allele in the control samples

^c^HRM analysis, High Resolution Melt analysis

^d^RFLP analysis, Restriction Fragment Length Polymorphism analysis
